# Research on Multirobot Pursuit Task Allocation Algorithm Based on Emotional Cooperation Factor

**DOI:** 10.1155/2014/864180

**Published:** 2014-07-23

**Authors:** Baofu Fang, Lu Chen, Hao Wang, Shuanglu Dai, Qiubo Zhong

**Affiliations:** ^1^School of Computer and Information, Hefei University of Technology, Hefei 230009, China; ^2^College of Electronic and Information Engineering, Ningbo University of Technology, Ningbo 315016, China

## Abstract

Multirobot task allocation is a hot issue in the field of robot research. A new emotional model is used with the self-interested robot, which gives a new way to measure self-interested robots' individual cooperative willingness in the problem of multirobot task allocation. Emotional cooperation factor is introduced into self-interested robot; it is updated based on emotional attenuation and external stimuli. Then a multirobot pursuit task allocation algorithm is proposed, which is based on emotional cooperation factor. Combined with the two-step auction algorithm recruiting team leaders and team collaborators, set up pursuit teams, and finally use certain strategies to complete the pursuit task. In order to verify the effectiveness of this algorithm, some comparing experiments have been done with the instantaneous greedy optimal auction algorithm; the results of experiments show that the total pursuit time and total team revenue can be optimized by using this algorithm.

## 1. Introduction

In recent years, multirobot task allocation problem has attracted widespread attention, which is widely used in robotic soccer, robotic rescue, intelligent warehouse, and other issues [1–3]. In order to solve the multirobot task allocation problem, Elango develops a simulation model involving task priority and the utilization of robots and refers to it as a balance multirobot prioritized task allocation (BMRPTA) problem [4]. This simulation model tries to find a balanced exploration path for each robot which is so constituted that the average waiting time and total completion time of all tasks are minimized. This allocation method assumes that robots are completely rational, so it does not consider the benefits of the individual robot. Auction [5, 6] is a fast and effective method of negotiation; when applying it into multirobot task allocation, robots can ensure the maximization of the overall benefits, at the same time considering their own benefits. Eric and Ofear [7] give up the ideas from traditional auction methods to pursue optimal task allocation strategy, so this paper focuses on the study of how to use robots efficiently in each team and how to prevent robots interfering with each other in the process of task execution, as well as how to execute the allocated tasks efficiently based on auction. Nanjanath and Gini [8] take more attention to the impact of robotic failures and environmental uncertainties in auction, so they aim at finding a compromise between computational complexity and quality of allocations and ability to adapt. If a robot finds an unexpected obstacle, experiences any other delay, loses communication, or is otherwise disabled, the rest of the team continues to operate. Above literatures take into account the self-interest of robot, but they do not have a deep research about self-interested robots such as the ability of self-interested robots, the individual cooperative willingness of robots in different situations, and the changes of individual cooperative willingness during executing tasks.

Psychology shows that emotion plays a very essential role in the process of work, where negative emotions may cause people's action to be slow and insensitive for the work while positive emotions can be conducive to the work [9]. Delgado-Mata and Ibanez-Martinez [10] add emotional mechanism to robots and use emotion to reflect the self-interest features of robots. When robots have negative emotions, such as sadness and fear, they are trended to keep the status quo and do not participate in the task; while robots have the positive emotion, they choose to participate in the task.

In order to measure individual cooperative willingness of self-interested robot in multirobot task allocation problem, a multirobot pursuit task allocation algorithm which is based on emotional cooperation factor is proposed. We build emotional cooperation factor for self-interested robots, then use it to measure the individual cooperative willingness of self-interested robots, and determine whether the robots are suited to participate in the pursuit task or not. Emotional cooperation factor is updated based on emotional attenuation and external stimuli. Combined with the two-step auction algorithm recruiting team leaders and team collaborators, set up pursuit teams and finally use certain strategies to complete the pursuit task.

## 2. Emotional Cooperation Factor

### 2.1. Definition

In order to describe the willingness intensity of the robot to participate in the pursuit, this paper enhances an emotion model constructed in our work before [11]. At first, the emotional cooperation factor *f*, representing the robots' emotional cooperative willingness, can be defined as follows:

(1) $$\begin{array}{c} f=I\times M_{t}\times \left(w_{1},w_{2},w_{3}\right), \end{array}$$

where the basic emotion status is described by a set *E* = {*e*_*i*_∣*i* = 1,2,…, *N*} and *e*_*i*_ is corresponding to happiness, anger, sadness, and joy; **I** is an emotional intensity vector; **I** = [**I**_1_, **I**_2_,…, **I**_**N**_]; **I**_**i**_ is the emotional intensity of basic emotion status *e*_*i*_. **M**_**t**_ is the exchanging matrix from basic emotion to PAD model where pleasure-arousal-dominance (PAD), three-dimensional model of emotion, is proposed by Chen and Long [12]. The value of *f* is decided by the type of the basic emotion status *e*_*i*_; *w*_*i*_(*i* = 1,2, 3) are the weights of PAD in each dimension where ∑_*i*=1_^3^ *w*_*i*_ = 1.

**Definition 1 .**

*f*_*c*_ is a justice factor for robot cooperation, which is calculated to judge whether the robot is willing to attend pursuit task allocation:

(2) $$\begin{array}{c} f_{c}=\{\begin{array}{cc} 1, & f>\xi \\ 0, & f\leq \xi , \end{array} \end{array}$$

where *ξ* is a justice parameter of the emotional cooperation factor. If the value of emotional cooperation factor is above *ξ*, the robots' emotion will be pleased and stable, whereas if the value of emotional cooperation factor is below *ξ*, the robot will be variable and unstable.

### 2.2. Several Impacts on Emotional Cooperation Factor

#### 2.2.1. Emotional Decay

Emotional intensity will decrease with the time passing by which directly causes the change of emotional intensity vector **I** and then influence the value of emotional cooperation factor. Based on the 3rd law of emotional intensity in psychophysics, the emotional decay curves comply with an exponential function *y* = *e* ^−*x*^. In order to simplify the process of emotional decay, we only consider previous emotional decay impact on the emotion of current moment. So the function of emotional decay can be expressed as follows:

(3) $$\begin{array}{c} \varphi \left(k_{i}\right)=e^{-k_{i}T},\\ I_{t}=I_{t-1}\times \varphi \left(k_{i}\right), \end{array}$$

where **I**_**t**−1_ is emotional intensity vector at *t* − 1; *T* is the checking period for emotional status; and *k*_*i*_ is an emotional decay factor.

#### 2.2.2. Emotional Stimuli

External stimulation is another important factor affecting the emotional status. Pursuing different evader can get different rewards. The higher reward given, the greater stimulation intensity produced. During the pursuit, if the robot is in depressed mood or emotion decay for pursuit unwilling to complete the task, we need to impose different stimulation intensity for different types of robots to ensure that the robot will complete its pursuit.

By using a stimuli vector *s* ^1^ *s* ^2^,…, *s* ^*r*^ to represent a real world stimulation, a stimuli with the kind of *i* and the intensity of *r* can be written as **S**_**i**_^**r**^ = *s*_*i*_^1^ *s*_*i*_^2^,…,  *s*_*i*_^*r*^ = *s*_*i*_ *s*_*i*_,…, *s*_*i*_ *s*,  *r* = 1,2,…, *r*_max⁡_, where *r*_max⁡_ is the maximum emotional intensity. By simulating robot's emotions and personality, Wang and Teng [13] define the matrix of stimulation **B**, assuming that stimulation is instantaneous without considering time; the emotional intensity can be expressed as follows:

(4) $$\begin{array}{c} \Phi \left(I_{i,t}\right)=I_{i,t}\times B. \end{array}$$

In summary, the value of emotional cooperation factor is influenced by the emotional decay and emotional stimuli. It can be transformed to the change of emotional intensity vector **I**, which can be defined as follows:

(5) $$\begin{array}{c} I_{i,t}=g\left(\overset{N}{\underset{i=1}{\sum }}\varphi \left(I_{i,t-1}\right)+\beta \Phi \left(I_{i,t}\right)\right), \end{array}$$

where *g* is a limiting function [14] that confines the value of emotional intensity within [0,1]; we set *g*(*x*) = 1/(1 + *e* ^−*x*^); *β* is the active threshold of stimuli and the value of *β* corresponds to the type of robot for stimulus; the fact that a different type of stimuli gives different effects of emotion changes can be expressed.

## 3. Multirobot Pursuit Task Allocation Algorithm

The contract net protocol is proposed by Smith [15] in 1980 at first and it is a typical market method which origins from the contractual mechanisms in the business activity. In the pursuit task allocation problems, emotional robots choose the appropriate evaders to pursue using contract net protocol. This paper provides a multirobot pursuit task allocation algorithm based on emotional cooperation factor to get appropriate pursuers and form the optimized cooperation teams at last.

### 3.1. Basic Definition

**Definition 2 .**

Define *E* = {*E*_1_, *E*_2_,…, *E*_*m*_} as a set of evaders, where *E*_*i*_ is represented by four elements *E*_*i*_ = 〈*P*_*e*_, *T*_*e*_, *C*_*e*_, *R*_*e*_〉, where *P*_*e*_ indicates the position of the evaders, represented by (*e*_*x*_, *e*_*y*_); *T*_*e*_ = {*t*_*i*_∣*i* = 1,2,…} is the type's set of the evader, which may have various types; set *C*_*e*_ = {*c*_*i*_∣*T*_*e*_ = *t*_*i*_,  *i* = 1,2,…} indicates the capability of each evader needed. Since the evaders have different types, and this means that catching evaders needs different type and different number of robots. Therefore, the key point to construct a pursuit team is to decide the number and the relative types of the pursuers. Define the reward after completing a pursuit task, *R*_*e*_ = {*r*_*i*_∣*T*_*e*_ = *t*_*i*_, *i* = 1,2,…}. The pursuer can gain different reward to capture a different type of evaders.

**Definition 3 .**

Define the pursuers set *P* = {*P*_1_, *P*_2_,…, *P*_*n*_}. *P*_*j*_ has five elements *P*_*j*_ = 〈*P*_*p*_, *T*_*p*_, *C*_*p*_, *E*_*p*_, *f*〉, where *P*_*p*_ is the position of the pursuer, indicated by (*p*_*x*_, *p*_*y*_); set *T*_*p*_ = {*t*_*i*_∣*i* = 1,2,…} represents the different type of the pursuer; set *C*_*p*_ indicates the capability of each pursuer, *C*_*p*_ = {*c*_*i*_∣*T*_*p*_ = *t*_*i*_, *i* = 1,2,…}, which embodies the characteristics of heterogeneous of different kinds of pursuers; *E*_*p*_ indicates the emotional intensity of the pursuers, which is a *N* dimensional vector *E*_*p*_ = (*I*_1_, *I*_2_,…, *I*_*N*_); *f* represents the value of emotional cooperation factor introduced before, which shows the willingness of the pursuer to complete its task.

**Definition 4 .**

Use *G* to represent the team gain after capturing an evader. After evader *E*_*i*_ is captured, the gain *G*_*i*_ of team *G*_*p*_*i*__ can be calculated by the cost of pursuit Cost_*i*_ and reward of the task *R*_*e*_*i*__, as follows:

(6) $$\begin{array}{c} G_{i}=R_{e_{i}}-{\text{Cost}}_{i}. \end{array}$$

During the pursuit, the cost of the team is mainly reflected on cost of distance Cost_*d*_*i*__ and emotional cost Cost_*e*_*i*__:

(7) $$\begin{array}{c} {\text{Cost}}_{{}_{i}}={{\text{Cost}}_{d}}_{{}_{i}}+{\text{Cost}}_{e_{i}}, \end{array}$$

where Cost_*d*___*i*__ is the sum of the distance between each team's robots of *G*_*p*_*i*__ and the target evader *E*_*i*_. Supposing *m* is the number of the pursuers in team *G*_*p*_*i*__, the cost of distance Cost_*d*___*i*__ can be written as follows:

(8) $$\begin{array}{c} {{\text{Cost}}_{d}}_{{}_{i}}=\overset{m}{\underset{j=1}{\sum }}\sqrt{{\left({p_{x}}_{{}_{j}}-e_{x_{i}}\right)}^{2}+{\left(p_{y_{{}_{j}}}-e_{y_{i}}\right)}^{2}}. \end{array}$$

The emotional cost Cost_*e*_*i*__ is carried out by its mood swings. In the stage of allocation, we need to predict the number of pursuers who may have emotional risk. Firstly calculate the time *t*_*j*_ that pursuer *P*_*j*_ needs to move to the evader, then estimate its emotional status after *t*_*j*_, and calculate the value of *f*_*j*_ in that time; if *f*_*j*_ < *ξ*, it means that this pursuer may have emotional risk; otherwise it does not. So the emotional risk of each pursuer can be represented to be as follows:

(9) $$\begin{array}{c} {\text{Cost}}_{e}=a\times r_{e}, \end{array}$$

where *a* is the number of robots who may have emotional risk and *r*_*e*_ is the emotional risk cost.

### 3.2. Each Pursuit Team Leader Confirmed

In this paper, hybrid architecture is used to assign the pursuit task. There is a central administrator and some distributed robots in the system. The functions of central administrator include collecting information of evaders, publishing evaders' information, receiving the bid, and authorizing contracts. The first auction is to recruit a leader for each pursuit team, who likes a practical project contractor. When the pursuers in the team are not enough, administrator takes charge of recruiting pursuers. The algorithm of recruiting team leader is as follows:

- The central administrator sends a recruiting message to robots, also giving them leaders' number *N*_*e*_ that the central administrator needs, and then publishes the detailed information of all evaders.
- Pursuer *P*_*j*_ receives the message, calculates its own *f*_*j*_, and sends it to the central administrator.
- The central administrator will count the number *N*_*p*_ of the robots which fit *f*_*j*_ ≥ *ξ* and then publish the leader they will recruit. After that, if *N*_*p*_ < *N*_*e*_, the administrator will send a stimulation command to each robot to update their *f*_*j*_. Pursuit robot *P*_*j*_ will accept the command and recalculate its *f*_*j*_ and then send it to the central administrator and then back to step b; if *N*_*p*_ ≥ *N*_*e*_, just continue.
- Pursuer *P*_*j*_ checks the evader information of each pursuit task and calculates its gain, then uses each gain as a bid value, and sends it back to the central administrator.
- Central administrator views the tenders submitted by pursuers and selects the most appropriate pursuer based on bid values for each task, so that all team leaders have the shortest total distance to the evaders. The problem can be transformed into a classic assignment problem; the Hungarian algorithm [16] is the most appropriate.
- The contract was awarded to the successful pursuing robots. After the pursuers receive the contract, the contract becomes effective. So it becomes the team *G*_*p*_*i*__'s leader, and then the team is formally established.

According to Definition 2, different capability is needed to capture each evader. If the leader is fully capable to catch the evader, it will do it alone. Otherwise, leader with insufficient capability needs other cooperators, so it should recruit other pursuers.

### 3.3. Recruiting Cooperators

At first, each insufficient leader sends its team's message to other free pursuers. Leader will choose appropriate members to join its team. The relative algorithm is listed below.

- Leaders will check whether their team's capability meets the requirement. If not, the leader will publish team's information to recruit collaborators including the evaders position, the capability value they still need, the reward, and the leader's information.
- Free pursuer *P*_*j*_ receives the recruitment information, calculates its *f*_*j*_, and sends it to the leaders with corresponding recruitment information.
- If the value of *f*_*j*_ is greater than *ξ*, which shows pursuer *P*_*j*_ is willing to cooperate with other robots, then the pursuer *P*_*j*_ count its gain as a bid value send to the leader.
- Leader views the tenders received and selects the most appropriate cooperators based on bid values for each team, so that all pursuit teams have the biggest total gains. The problem can also be transformed into a classic assignment problem, solved by the Hungarian algorithm [16].
- Each leader counts the total capability *C*_*g*_*i*__ after new cooperator attends in its team, if *C*_*g*_*i*__ ≥ *C*_*e*_*i*__, where *C*_*e*_*i*__ is an total capability to complete the task, the team can be established; whereas it not meet the capability requirement, the leader sends a emotional stimuli to the pursuer, then jump to step a.
- When all of the teams have enough capability as the task required, an allocation matrix *Q* will be generated and contracts will be sent from the leader to its cooperators. If some teams still need more cooperators to complete the task while there are no free robots, they must wait until the eligible robot completes its task.

After the contracts are received by cooperators, the pursuit team *G*_*p*_ will be established.

### 3.4. Pursuit Strategy

In order to verify the effectiveness of multirobot task allocation algorithm proposed by this paper, we combine this multirobot task allocation algorithm with some pursuit algorithm to complete the pursuit task. We use the pursuit algorithm as follows.

Assume that there is a virtual force field in the pursuing process. The force from pursuers to the evader is repulsion, whereas that from evader to pursuers is an attraction. The pursuers will move according to the attraction and the evader will run depending on the repulsion. We assume that the size of attraction and repulsion is related to distance. Attraction *F*_*a*_ and repulsion *F*_*r*_ can be defined as follows:

(10) $$\begin{array}{c} F_{a}=\gamma \frac{1}{E_{d_{i}}},\\ F_{r}=\gamma \left(\frac{1}{P_{d_{1}}}+\frac{1}{P_{d_{2}}}+\cdots +\frac{1}{P_{d_{n}}}\right), \end{array}$$

where *γ* is scale factor; *E*_*d*_*i*__ is the distance from the pursuer to the evader *E*_*i*_; *P*_*d*_*i*__ is the distance from each pursuer in pursuit team to its own evader. Draw a unit circle which uses the position of evader to be the center of the circle and then split the circle into *h* parts on the circumference; the direction of the evader will be chosen from the *h* directions which come from the center to the *h* dividing points. Calculate the repulsion *F*_*r*_ of the evader to get to each of the divisions; choose the direction with the least repulsion *F*_*r*_ as the motion direction. In the same way, choose the direction with the maximum attraction *F*_*a*_ as the motion direction for pursuit.

**Definition 5 .**

Use *f*_*d*_ as the capturing distance judgment factor which can predict whether evader is captured or not:

(11) $$\begin{array}{c} f_{d}=\{\begin{array}{cc} 1, & \exists E_{d_{i}}\leq \kappa \\ 0, & \forall E_{d_{i}}>\kappa , \end{array} \end{array}$$

where *κ* is the available distance for capture; when the distance between pursuer and evader is below *κ* in the same pursuit team, it means that this evader is captured.

## 4. Experiments

### 4.1. Relevant Parameters Setting

In the experiments, we let *N* = 3 and select dread, anger, and joy as three kinds of emotion as a basic emotion. According to the paper [11], we get the transformation matrix from basic emotion to PAD emotion model which showed the following:

(12) $$\begin{array}{c} M_{t}=\left[\begin{array}{ccc} -0.64 & 0.60 & -0.43\\ -0.51 & 0.59 & 0.25\\ 0.40 & 0.20 & 0.15 \end{array}\right]. \end{array}$$

From the paper [14], we find that “dominance” in the PAD model has the maximum impact on emotional cooperation factor, “pleasure” follows, and “arousal” has the minimal impact. Therefore, respectively, set *w*_1_ = 0.2, *w*_2_ = 0.1, *w*_3_ = 0.7. With the passage of time, emotional intensity infinitely tends to zero, so we select *ξ* = 0.001. The pursuer is not willing to participate in pursuit, when *f* < 0.001. On the contrary (*f* > 0.001), it means the pursuer is willing to finish the task.

**Figure 1:**
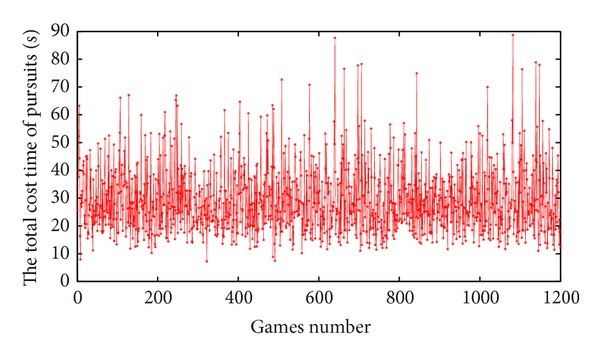
1200 sets of experiments without emotional decay.

Define *n* = 12,  *m* = 4, so 12 pursuers pursue 4 evaders and produce the initial position of evaders and pursuers *t* in the 100 × 100 square area. In order to determine the influence of emotional decay factor *k* experiments, we have randomly done 1200 sets of experiments without emotional decay; the experimental results are shown in Figure 1.

**Figure 2:**
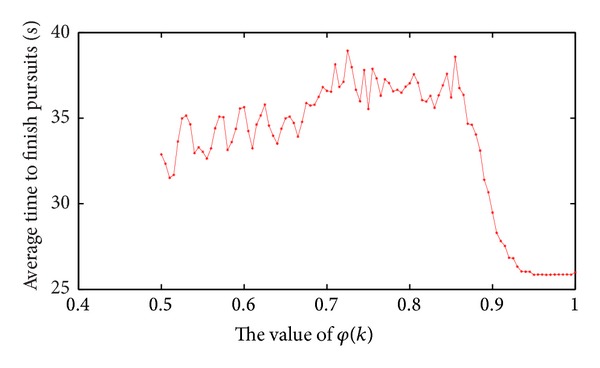
The effect of *φ*(*k*) on pursuing time.

From Figure 1, the total cost time of pursuit from 20 to 30 seconds is more intensive, so we randomly select 100 sets of data from the 1200 sets of initial data which obtained from above experiments, then use the 100 sets of data for the next experiments. For the following experiment, suppose emotional decay types of robots were the same in all experiments; namely, emotion decay factor *k* was the same. Set *T* = 0.1, so check the emotional status of pursuit robot every 0.1 second. When the value of *φ*(*k*) is too small, emotional decay will be too fast, not conducive to experiment, so the value of *φ*(*k*) is fixed between 0.5 and 1. Using the 100 groups' initial data, experiments are carried out under different values of *k*; then obtain the total cost time of pursuit; finally, calculate the average time to finish all pursuits by different *φ*(*k*). Result is shown in Figure 2.

Figure 2 shows that when the value of *φ*(*k*) is between 0.5 and 0.85, average time has larger effect by *φ*(*k*). With the increase of *φ*(*k*), average time shows a trend of increase, but it has greater volatility. When *φ*(*k*) is between 0.85 and 0.93, pursuit time decreases and *φ*(*k*) decays obviously. When the value of *φ*(*k*) is between 0.93 and 1, it has little effect on pursuit time. To ensure the stability of experiment and reflect the impact of *φ*(*k*) to pursuit time, we control the value of *φ*(*k*) between 0.85 and 0.93, so set *φ*(*k*) = 0.87, so emotional decay factor *k* = 1.39.

In the pursuit strategy, the forces of attraction and repulsion are just the parameter to determine the motion direction for evaders and pursuers, whose value is too large or too small having no effect on determining the actual motion direction. In order to simplify the experiment we set *γ* = 1. Choose *h* = 36; evaders select the direction that will produce the smallest repulsive force. Take *κ* = 0.8; when the distance between pursuit robot and evader is less than or equal to 0.8 m, that means the evader has been captured.

### 4.2. Experiment and Data Analysis

**Figure 3:**
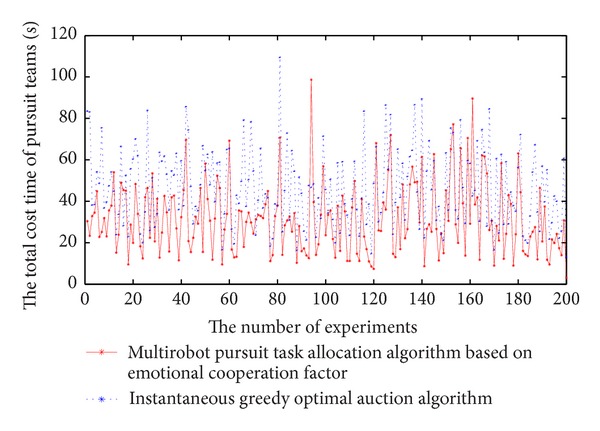
The comparison chart of total cost time of pursuit.

**Figure 4:**
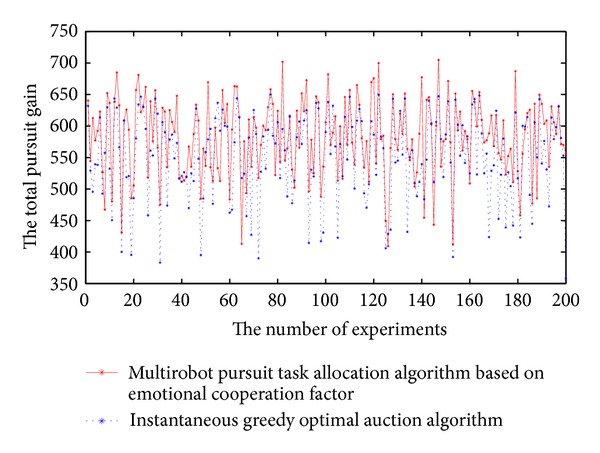
The comparison chart of total gain for pursuing teams.

To verify the effectiveness of the algorithm in this paper, we have made a comparison with the method of instantaneous greedy optimal auction algorithm [17]. The 200 experiments are designed under the same scene. As Figures 3 and 4 show, comparing with instantaneous optimal auction greedy algorithm in the same scenario, 86.5% total time of pursuit can be shortened and 77.5% total gain is higher. When use instantaneous greedy optimal auction algorithm to establish the pursuit teams, all robots are self-interested, so every robot would like to choose joining the team which gives itself the greatest gain, thus self-interested robots may lead to part of teams' capacity excess and another part of teams lack of capacity, it is not conducive to pursuit task allocation. In our algorithm, all pursuers have emotions, and they consider personal gain while taking into account the global gain for pursuit team. At the stage of pursuit task allocation, when pursuers meet conflict between global gain and their own gain, in order to obtain greater global gain they will sacrifice their own gain. So our algorithm can optimize the total pursuing time and global gains. Analyzing the experiments with poor total pursuit time or global gains, we found that the initial position of robots is very favorable to instantaneous greedy optimal auction algorithm.

## 5. Conclusions

In this paper we have a deep research about self-interested robots, such as the ability of self-interested robots, the individual cooperative willingness of robots in different situations, and the changes of individual cooperative willingness during executing tasks. We proposed a multirobot pursuit task allocation algorithm based on emotional cooperation factor. In order to measure individual cooperative willingness of self-interested robot in multirobot task allocation problem, emotional cooperation factor, a value updated based on emotional attenuation and external stimuli in the process of pursuit is introduced into emotional robot. By using two steps of auction for allocation, several subteams and their leaders and cooperators are selected and then choose a certain pursuit strategy to complete pursuit task. As the experiment shows, our algorithm optimizes the cost of pursuit time and total team's gain, as well as proving the effectiveness.
